# Longitudinal association of adiposity with wheezing and atopy at 22 years: the 1993 Birth Cohort, Pelotas, Brazil

**DOI:** 10.2147/JAA.S183699

**Published:** 2018-11-30

**Authors:** Ana Maria Baptista Menezes, Paula Duarte de Oliveira, Cauane Blumenberg, Efrain Sanchez-Angarita, Gloria Isabel Niño-Cruz, Ignacio Zabert, Janaina Calu Costa, Luiza Isnardi Cardoso Ricardo, Rafaela Costa Martins, Fernando César Wehrmeister

**Affiliations:** 1 Postgraduate Program in Epidemiology, Federal University of Pelotas, Pelotas, RS, Brazil, anamene.epi@gmail.com; 2 Pulmonary Department, Hospital Universitario de Caracas, Universidad Central de Venezuela, Capital District, Venezuela; 3 Facultad de Medicina, Universidad Nacional del Comahue, Neuquén, Argentina

**Keywords:** asthma, BMI, fat mass, fat mass index

## Abstract

**Purpose:**

Asthma is a highly prevalent noncommunicable lung disease. The aim of this study was to evaluate the longitudinal association of obesity/adiposity with wheezing and atopy.

**Methods:**

The population of the study was composed of participants from the 1993 Pelotas (Brazil) Birth Cohort. The following outcome variables were measured at 22 years: wheezing in the last 12 months, wheezing with atopy, wheezing without atopy, only atopy, and persistent wheezing at 18 and 22 years. Exposure variables were obesity body mass index, percent fat mass (FM), and fat mass index, which were obtained by precise methods (BOD POD and dual-energy X-ray absorptiometry [DXA]). Crude and adjusted logistic and multinomial logistic regressions were used in the analyses.

**Results:**

The prevalence of wheezing (with and without atopy), wheezing without atopy, only atopy, and persistent wheezing were 10.6%, 3.9%, 30.9%, and 4.0%, respectively. To be obese or to belong to the highest tertile of obesity/adiposity at two follow-ups showed a cumulative and positive association with wheezing in the adjusted analysis; for atopy there was no significant association. The odds ratio (OR) for wheezing according to the percentage of total FM measured by DXA in the highest tertile at both follow-ups was 1.58 (95% CI: 1.14–2.20) against an OR of 1.16 (95% CI: 0.92–1.47) for atopy. Persistent wheezing was also associated with adiposity, but without statistical significance.

**Conclusions:**

We found a positive longitudinal association between several measures of adiposity and wheezing at 22 years old. The effect was higher for cumulative adiposity; the results for atopy were not consistent.

## Background

Most of the literature shows a direct association between asthma or wheezing and obesity.^1^–^4^ Two systematic reviews among children^5^,^6^ showed that overweight and obesity are associated with an increased risk of childhood asthma. Besides the systematic reviews among children, a systematic analysis among adults showed that asthma incidence increases by 50% in overweight/obese subjects with a dose–response relationship without effect of gender.^7^

Although there is almost a consensus about the association of asthma/wheezing and obesity, few studies have explored the atopic and nonatopic phenotypes of asthma, and the results are not so consistent as the previous ones. In a systematic review for risk factors for nonatopic asthma/wheeze in children and adolescents, obesity was found to be a risk factor.^8^ However, the majority of the studies have used body mass index (BMI) or skinfolds as the main measure of obesity and these indicators are not the best methods for measuring fat mass (FM) or lean mass; it has been recognized that BMI does not reflect adiposity and the gold standard for adiposity is measured by dual-energy X-ray absorptiometry (DXA) or by BOD POD.^9^,^10^

Most longitudinal studies on this subject have been carried out outside Latin America (LA), and it is plausible to think that findings in LA can be different from developed countries because the prevalence of atopy is very high in LA.^11^

Based on this, the aim of this paper was to measure the longitudinal association of obesity and adiposity measured by BMI, DXA, and BOD POD with three phenotypes of asthma: wheezing, atopy, and persistent wheezing, in a birth cohort from Southern Brazil, at the beginning of adulthood.

## Methods

In 1993, all hospitals in the city of Pelotas were visited daily from 1 January to 31 December 1993. All mothers who had given birth at these institutions were invited to take part in the study and soon after delivery they were interviewed.^12^ From the 5,265 live births in the city, 5,249 were enrolled in our birth cohort study. Subsamples of the cohort were followed up during childhood,^12^ and all cohort members were sought when they had reached the mean age of 11, 15, 18, and 22 years.^13^

The outcomes were defined according to the questionnaire of the “International Study of Asthma and Allergies in Childhood Steering Committee”, which has already been validated in Brazil^14^,^15^: a) presence of wheezing in the last year collected at 22 years, b) wheezing without atopy at 22 years, c) wheezing with atopy, and d) only atopy symptoms. The outcome “persistent wheezing” (presence of wheezing at 18 and at 22 years) was also analyzed and it has been shown as Table S1. Wheezing was treated as a binary variable and considered present after a positive answer to the following question: “Have you had wheezing or whistling in the chest in the past 12 months?”. Atopy symptoms were assessed based on binary questions about rhinitis and eczema: 1) “In the last 12 months, have you had any problems with sneezing, runny nose, or stuffy nose without having a flu or a cold?”; 2) “Have you ever had itchy rash, which have appeared and disappeared for at least 6 months? 3) In the last 12 months, have you had these itchy rashes on your skin?”; If the participant answered yes for any of the three questions, we considered that he had atopy symptoms in the last year.

The main exposure variables, collected at 18- and 22-year follow-ups, were BMI (kg/m^2^), percentage of total FM measured by the BOD POD (air displacement plethysmography – BOD POD^®^ Composition System; COSMED, Albano Laziale, Italy),^16^ percentage of segmented (trunk) FM measured by DXA – model Lunar Prodigy Advance^®^; GE Healthcare Europe GmbH, Freiburg, Germany), and fat mass index (FMI; fat kg [BOD POD]/height m^2^). BMI was categorized as obese (≥30 kg/m^2^),^17^ and percentage of FM (according to BOD POD), percentage of total and trunk FM (according to DXA) and FMI as tertiles. Also, four variables were generated classifying individuals according to BMI, total FM, trunk FM, and FMI as follows: none – reference category – (no obese or not in the highest tertile either at 18 or at 22 years), obese or belong to the highest tertile only at 18 years, obese or belong to the highest tertile only at 22 years, and obese or belong to the highest tertile at both ages (18 and 22 years).

Categorical variables were described as absolute and relative frequencies, whereas continuous variables were described by mean and SDs. Crude and adjusted logistic regression and multinomial logistic regression were used to test associations between the outcomes and exposures.

Logistic regression was used for wheezing in the last year and for persistent wheezing, while multinomial regression was used for wheezing and atopy combined (only wheezing, wheezing without atopy, wheezing with atopy, and only atopy); the reference category was those who did not report wheezing and/or atopy. For adjustment, the following covariates were taken into account: sex (male/female), self-reported skin color (white/black/brown/other), asset index (quintiles), years of study (complete years), maternal smoking during pregnancy collected at birth (yes/no), variables that had been gathered at the 11-year follow-up (parents smoking during childhood, parents history of asthma, wheezing or bronchitis, hospitalization during the first 10 years), household crowding (number of house residents) collected at 18 years follow-up; smoking and physical activity at leisure and/or commuting at 18 and 22 years (categorized as none, present only at 18, present only at 22, and present at both follow-ups). Physical activity was measured through a standardized and previously tested questionnaire (IPAQ)^18^ (categorized as active for ≥300 and 150 minutes a week, according to cutoff points for adolescent and adults, respectively). Confounders were chosen a priori according to previous knowledge and according to the available information on the cohort. Interaction between body composition exposures and sex was tested but it was not significant; therefore, we opted to show results adjusted to sex but nonstratified. Significance level was set as 5% and all analyses performed using Stata 13.1 software (Stata Corp LP, College Station, TX, USA).

Interviewers underwent standardization testing before beginning of field work and every 2 months afterward to determine repeatability and validity of weight, height, and skinfold measurements. All cohort follow-up projects were approved by the Federal University of Pelotas Ethics Committee. The protocols for the 18- and 22-year visits were 05/11 and 1.250.366, respectively. The cohort participants, or their caregivers, signed the term of free and informed consent prior to participation and the study was conducted in accordance with the Declaration of Helsinki.

## Results

The response rate of the 1993 Birth Cohort at 11, 15, 18, and 22 years was 87.5%, 85.7%, 81.4%, and 76.3%, respectively.^13^ The characteristics of the 3,810 members of the 1993 Pelotas Birth cohort followed up when they were 22 years old (Table 1). Most of them had white skin color and around 70% of them had nine or more years of schooling. Smoking during pregnancy was present in nearly one-third of the mothers and around 40% among fathers. Hospitalization during childhood was referred by 37% and family history of asthma, wheezing, or bronchitis by nearly 35%. The number of house residents was six or more people for 17% of the cohort participants at the 18-year follow-up. At 18 and 22 years, 44% of them were active and 9.9% were smokers. Obesity at both ages was 7.8% and the percentage in the highest tertile of all FM measures and of FMI at 18 and 22 years was similar (around 24.0%).

**Figure 1:**
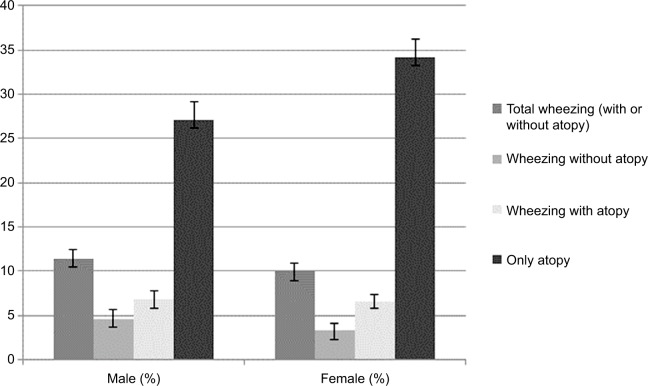
Prevalence of self-reported wheezing and atopy symptoms in the last 12 months at 22 years by sex. **Note:** The 1993 Pelotas Birth Cohort.

Figure 1 shows a prevalence of wheezing in the last 12 months of 10.6% (95% CI: 9.6–11.6), without significant difference according to sex (*P*=0.130) at the age of 22 years, while wheezing without atopy was more prevalent among males (4.6%; 95% CI: 3.6–5.6) than females (3.3%; 95% CI: 2.5–4.1) (*P*=0.04). The highest prevalence was for the outcome “atopy symptoms” reaching 30.9% (95% CI: 29.5–32.4) and more frequent among females (34.3%; 95% CI: 32.2–36.3) than males (27.1%; 95% CI: 25.1–29.2). Persistent wheezing, that is, those who have answered yes for wheezing at 18 and at 22 years old, did not differ according to sex (4.0%; 95% CI: 3.3–4.6) (data shown as Table S1).

Mean BMI was slightly higher at 22 than at 18 years and similar according to sex. The percentage of FM measured by BOD POD and DXA was higher at 22 compared to 18 years and higher among women than men at both follow-ups; the same pattern was observed for FMI (Table 2).

Obesity and most measures of adiposity were positively associated with wheezing at 22 years (Table 3). In the adjusted analysis, for those who were obese at 18 and 22 years or did belong to the highest tertile of FM and FMI also at both follow-ups, the odds ratio (OR) for “wheezing” was higher than for those who were not obese or did not belong to the highest tertile of adiposity. Also, the ORs for wheezing in the last 12 months varied from 2.0 (95% CI: 1.32–3.03), for those who were obese at both follow-ups, to 1.58 (95% CI: 1.14–2.20) for those belonging to the third tertile of percentage of total FM at both ages.

Very similar results were observed between obesity and measures of adiposity for the outcome “only wheezing without atopy”, with the highest ORs for those who had been obese or who belonged to the highest tertile of adiposity at 18 and at 22 years, in the adjusted analysis. Although the OR was 1.59 for wheezing without atopy according to FMI in the adjusted analysis at both follow-ups, the confidence interval included the unit (Table 3). For wheezing plus atopy (outcome shown in the third column of Table 3), there was an association between the highest tertile of total FM measured by DXA and trunk FM (OR of 1.60; 95% CI: 1.06–2.42; OR of 1.65; 95% CI: 1.08–2.51) with *P*-values of 0.021 and 0.014, respectively. For the outcome “only symptoms of atopy”, in the adjusted analysis there was a positive and significant association only for the exposure “percentage trunk FM” at both follow-ups (OR=1.26; 95% CI: 1.0–1.59) (Table 3). Persistent wheezing followed the same pattern as wheezing, with higher OR for those who were obese or in the third tertile of adiposity at both ages (18 and 22 years), although probably due to the sample size the results did not reach statistical significance (Table S1)

## Discussion

This paper describes a positive association between cumulative obesity/adiposity at 18 and 22 years and the phenotype wheezing at 22 years, even after adjustment for confounders; this association seems to be more due to wheezing than to atopy, because the results for the atopic phenotype (alone) were not so consistent. Persistence of wheezing at both ages also showed the same pattern as for the phenotype wheezing, although there was absence of statistical significance due to lack of power.

Most of the literature has shown an association between wheezing and obesity; however, few articles have been tried to disentangle the different phenotypes (wheezing and atopy). Classification of phenotypes is important to predict the prognosis and response to treatment and understand its underlying mechanisms.^19^ Epidemiologic studies have considered instability of asthma phenotypes over time^20^,^21^ and identified associated risk factors for each asthma phenotype.^19^ Therefore, the aim of the present study was to evaluate the association of obesity/adiposity considering different phenotypes, such as atopic, nonatopic, and persistent.

It is plausible to think that symptoms of wheezing and atopy are a good proxy for asthma diagnosis, even without methacholine tests, because wheezing is the main symptom of asthma.^22^,^23^ One can argue this as a limitation of the paper, but at the young age of 18 and 22 years, asthma is the most prevalent chronic noncommunicable disease with wheezing as its main symptom; COPD is an important differential diagnosis from asthma, but COPD usually shows symptoms after the age of 40 years and not at young adulthood and its mains symptoms are cough, sputum, and dyspnea. The wheezing information may suffer from recall bias; however, we applied a well-known validated instrument to measure asthma symptoms, which eases such bias.^14^,^15^ Atopy in the present paper was based only on symptoms of rhinitis or symptoms of eczema, without skin prick tests or IgE measurement; this is indeed a limitation in the interpretation of the results, although it has been used in several studies.^24^,^25^

On the other hand, several strengths should be emphasized in this study. The prospective design allowed us to evaluate temporality and the cumulative role of the exposures for the studied outcomes. According to our results, it seems that current exposure (22 years) and cumulative exposure of obesity and adiposity (18 and 22 years) are the main risk factors for the wheezing phenotype at early adulthood, since the highest OR was observed for those subjects obese or with adiposity at two points in time (18 and 22 years) or for those with the exposure at 22 years. The very high attrition rates at all phases of this cohort reassure us of the robustness of the findings. Due to the low refusal rates and to the several follow-ups of this cohort, we were able to adjust for important potential confounders, which could be misleading the findings. Socioeconomic (SE) status is one of the most important confounders in the association between obesity/adiposity and wheezing and atopy; in this cohort it has been found that wheezing is more prevalent among poor people (12.7% in the poor tertile of income) than among rich people (9.3%), at 22 years (data not shown); and the prevalence of atopy shows the opposite pattern, prevalence of 5.6% among poor and 18.5% among rich, at the same age. Similar finding was observed in a historical cohort from Glasgow at adolescence and young adulthood age (16–30 years)^26^; they evaluated the effect of SE position based on several indicators and wheezing and atopy and it has been found that a low SE position was associated with wheezing and a high SE position was associated with atopy. Other study with the Avon Longitudinal Study of Parents and Children (ALSPAC) cohort at childhood (7–8 years) found an association between wheezing with low SE status and atopy with high SE status.^27^

Most studies have evaluated obesity only by BMI; however, BMI alone may not adequately characterize the relation between overweight or obesity and complex diseases such as asthma.^3^ For instance, its usefulness and predictive value have been questioned in studies of cardiovascular disease and diabetes.^28^,^29^ Adiposity measured by highly precise methods (BOD POD and DXA) – as it was performed in the present cohort – enabled us to analyze the % of FM and the FMI, which are better measures of adiposity than BMI.^9^,^10^

A very interesting study from a developed country (England) investigated whether the magnitude of the obesity/adiposity effect varied when asthma was classified as atopic and nonatopic; the study was based on the ALSPAC cohort at the age of 7–9 years, with measures of FM obtained by DXA.^4^ Using a Mendelian randomization approach, the authors observed a stronger effect of obesity/adiposity for nonatopic children (relative ratio [RR]: 1.90, 95% CI: 1.19–3.03) than for atopic according to BMI (RR: 1.37, 95% CI: 0.89–2.11) and according to FM an RR of 1.73 (95% CI: 1.17–2.55) and 1.25 (95% CI: 0.86–1.80), respectively.

Noal et al using data from the 1993 Birth Cohort from Pelotas found a positive association between wheezing and obesity measured by BMI and skinfolds during adolescence.^1^ The highest RR was found for prevalence of wheezing among those who had been obese at two follow-ups (11 and 15 years) with an adjusted RR of 1.44 (95% CI: 1.01–2.07); for those ever in the highest tertile of skinfolds trajectory from 11 to 15 years the adjusted RR was 1.34 (95% CI: 0.94–1.92); they also found an association between the same exposure categories and persistent wheezing (present at 11 and 15 years). The results of the analysis at 18 and 22 years in the Pelotas cohort reveal the same pattern of results as it had been observed at the beginning of adolescence; besides that, we have shown this association classifying the sample on the nonatopic (wheezing) and atopic phenotypes, and using a more accurate measure of adiposity such as BOD POD and DXA.

Similar results to our findings were also observed in the NHANES population (2–19 years), although they have measured obesity only by BMI; among nonatopic children and adolescents the OR for wheezing was 2.20 (95% CI: 1.15–4.22) and for atopic population there was not greater risk for those obese compared to nonobese.^2^

In a population-based study in Puerto Rican children (6–14 years), the authors found that adiposity indicators were associated with asthma, severity of asthma, and atopy, and they concluded that atopy was a mediator in this association through the mediation analysis^3^; the main limitation of this study was the cross-sectional design and the possibility of reverse causation since temporality could not be ascertained.

Some studies have shown an association between persistence or severity of asthma (atopic and nonatopic) and obesity; Noal et al found a positive association in the same cohort as the present one, at 11 and 15 years; the outcome was “wheezing in the last 12 months” (not specifically atopic and nonatopic); an RR of 1.79 (95% CI: 1.20–2.68) and 1.82 (95% CI: 1.10–3.02) for those adolescents obese and for those in the highest tertile of skinfolds at 11 and 15 years, respectively.^1^ In the NHANES study they found an OR of 2.14, although without statistical significance, from those who have had more medical visits due to wheezing among nonatopic than the atopic asthmatics (OR=1.10; 95% CI: 0.60–2.00).^3^ We subclassified the persistent wheezing phenotype in those only with “persistent wheezing” (without atopy) and those with “persistent wheezing and atopy” and we did not observe differences among these two phenotypes and obesity/adiposity (data not shown).

Several plausible mechanisms have been proposed to explain the relationship between obesity/adiposity and asthma, including enhanced systemic inflammation.^30^,^31^ Results from the NHANES study evaluating CRP suggest that overweight may indeed lead to systemic inflammation that in turn leads to an increased risk of asthma in nonatopic individuals. There was no evidence of this relationship between systemic inflammation and asthma among atopic youth according to NHANES data. These findings suggest that allergic and systemic inflammation may operate independently on the pathway to asthma.^2^

Another possible link would be through promotion of allergic inflammation by adipokine effects on the immune system,^32^ but our results and also those from other studies showing stronger associations of obesity with nonatopic,^4^ and no evidence that obesity is associated with atopy, lead us to conclude that the role of inflammation in the different phenotypes is currently unclear. We performed some analysis to see whether IL-6 and PCR were associated with atopic or nonatopic asthma in our cohort and we found a higher OR for a mean IL-6 in the third tertile at 18 and at 22 years for the phenotype wheezing and not for atopy, although most of the results were not statically significant; the OR for IL-6 and for CRP with atopy was protective (data not shown).

Given conflicting findings from studies of overweight or obesity (largely assessed by BMI) and atopy or nonatopic diseases, the role of inflammation in the different phenotypes is currently unclear.^2^,^33^–^35^

As the prevalence of obesity and asthma has been increasing over the last years in LA, and to the conflicting results of the association between the different phenotypes (atopic and nonatopic asthma) and obesity/adiposity, we suggest more studies to be carried out with a prospective design and using precise methods for measuring atopy and adiposity.

## Conclusion

There is a positive longitudinal association between cumulative obesity and adiposity at 18 and 22 years and wheezing at 22 years, even after adjustment for confounders; this association seems to be more due to wheezing than atopy, because the results for atopy were not so consistent.

## Supplementary material

**Table S1:** Association between persistent wheezing and body composition at 18 and 22 years

	Persistent wheezing (18 and 22 years/with or without atopy) ^a^	

	Crude	Adjusted
18 years/22 years	OR (95% CI)	OR (95% CI)

**BMI obese (≥30 kg/m ^2^)**	*P*=0.413	*P*=0.671
None	1.00	1.00
Only at 18 years	1.11 (0.34, 3.57)	1.50 (0.44, 5.05)
Only at 22 years	0.74 (0.37, 1.48)	0.77 (0.36, 1.64)
Both	1.50 (0.86, 2.61)	1.28 (0.66, 2.51)
**Total fat mass – BOD POD % in the third tertile**	*P*=0.923	*P*=0.937
None	1.00	1.00
Only at 18 years	1.11 (0.61, 2.02)	0.93 (0.43, 2.03)
Only at 22 years	0.88 (0.45, 1.73)	1.18 (0.57, 2.46)
Both	1.10 (0.72, 1.66)	1.12 (0.68, 1.83)
**Total fat mass – DXA % in the third tertile**	*P*=0.810	*P*=0.645
None	1.00	1.00
Only at 18 years	1.15 (0.60, 2.22)	0.99 (0.43, 2.26)
Only at 22 years	0.89 (0.44, 1.81)	0.89 (0.37, 2.15)
Both	1.20 (0.77, 1.85)	1.35 (0.82, 2.22)
**Trunk fat mass – DXA % in the third tertile**	*P*=0.764	*P*=0.472
None	1.00	1.00
Only at 18 years	1.26 (0.67, 2.37)	1.02 (0.45, 2.34)
Only at 22 years	1.21 (0.64, 2.28)	1.39 (0.66, 2.93)
Both	1.22 (0.78, 1.90)	1.46 (0.88, 2.42)
**FMI in the third tertile**	*P*=0.828	*P*=0.905
None	1.00	1.00
Only at 18 years	1.21 (0.66, 2.22)	1.08 (0.50, 2.32)
Only at 22 years	0.81 (0.40, 1.63)	0.84 (0.37, 1.90)
Both	1.04 (0.68, 1.58)	1.14 (0.70, 1.84)

**Notes:** The 1993 Pelotas Birth Cohort.

a Logistic regression (persistent wheezing: wheezing in the last 12 months reported at 18- and 22-year follow-ups – yes/no). Adjusted by gender, skin color, asset index (quintiles), education (years), maternal smoking during pregnancy, parents smoking during infancy, hospitalization during infancy, parents history of asthma, wheezing or bronchitis, household crowding, smoking trajectory 18–22 years, and physical activity trajectory 18–22 years.

**Abbreviations:** BMI, body mass index; DXA, dualenergy X-ray absorptiometry; FMI, fat mass index.

## Figures and Tables

**Table 1:** Characteristics of the 22-year follow-up sample

Variables	N (%)

**Gender**	
Male	1,783 (46.8)
Female	2,027 (53.2)
**Skin color**	
White	2,262 (63.3)
Black	538 (15.1)
Brown	637 (17.8)
Other	137 (3.8)
**Asset index (quintiles**)	
First (poorest)	761 (20.0)
Second	761 (20.0)
Third	761 (20.0)
Fourth	761 (20.0)
Fifth (richest)	760 (20.0)
**Education (years**)	
0–4	111 (2.9)
5–8	1,013 (26.6)
9–11	1,560 (41.0)
≥12	1,121 (29.5)
**Maternal smoking during pregnancy**	1,242 (32.6)
**Maternal smoking at 11 years follow-up**	
Never	1,693 (46.9)
Former	787 (21.8)
Smoker	1,131 (31.3)
**Paternal (or mothers partner) smoking at 11 years follow-up**	
Never	1,030 (37.2)
Former	707 (25.5)
Smoker	1,031 (37.3)
**Hospitalization during the first 10 years of life**	1,339 (37.1)
**Parents history of asthma, wheezing, or bronchitis at 11 years follow-up**	1,236 (34.8)
**Household crowding at 18 years**	
≤3	1,359 (38.6)
4–5	1,569 (44.5)
≥6	595 (16.9)
**Active at leisure and/or commuting at 18 and 22 years**	
None	648 (18.5)
Only at 18 years	569 (16.2)
Only at 22 years	749 (21.3)
Both ages	1,542 (44.0)
**Smoker at 18 and 22 years**	
None	2,824 (80.3)
Only at 18 years	117 (3.3)
Only at 22 years	229 (6.5)
Both ages	347 (9.9)
**BMI obese (≥ 30 kg/m^2^) at 18 and 22 years**	
None	2,689 (79.9)
Only at 18 years	69 (2.1)
Only at 22 years	344 (10.2)
Both ages	262 (7.8)
**Total fat mass – BOD POD % in the third tertile**	
None	1,905 (58.4)
Only at 18 years	302 (9.3)
Only at 22 years	287 (8.8)
Both ages	770 (23.6)
**Total fat mass – DXA % in the third tertile**	
None	1,751 (58.7)
Only at 18 years	259 (8.7)
Only at 22 years	270 (9.1)
Both ages	704 (23.6)
**Trunk fat mass – DXA % in the third tertile**	
None	1,741 (58.3)
Only at 18 years	271 (9.1)
Only at 22 years	280 (9.4)
Both ages	697 (23.3)
**FMI in the third tertile**	
None	1,910 (58.6)
Only at 18 years	273 (8.4)
Only at 22 years	278 (8.5)
Both ages	801 (24.6)

**Notes:** The 1993 Pelotas Birth cohort (n=3,810). Maximum missing values for DXA variables (total n=3,325).

**Abbreviations:** BMI, body mass index; DXA, dual-energy X-ray absorptiometry; FMI, fat mass index.

**Table 2:** Mean BMI and body composition variables at 18- and 22-year follow-up by sex

Variables	Males		Females	
	18 years Mean (SD)	22 years Mean (SD)	18 years Mean (SD)	22 years Mean (SD)

BMI (kg/m^2^)	23.3 (4.1)	25.1 (4.8)	23.5 (4.8)	25.6 (5.8)
Total fat mass (BOD POD %)	16.7 (8.7)	20.9 (9.8)	32.8 (7.8)	35.8 (8.5)
Total fat mass (DXA %)	16.8 (9.3)	20.8 (10.0)	34.9 (8.4)	38.0 (8.8)
Trunk fat mass (DXA %)	18.8 (10.5)	24.2 (11.5)	36.0 (9.3)	39.4 (9.7)
FMI (fat kg/height m^2^)	4.2 (3.0)	5.6 (3.7)	8.0 (3.6)	9.5 (4.4)

**Notes:** The 1993 Pelotas Birth Cohort. BMI n=3,588; BOD POD n=3,559; DXA n=3,325.

**Abbreviations:** BMI, body mass index; DXA, dual-energy X-ray absorptiometry; FMI, fat mass index..

**Table 3:** Association between body composition at 18 and 22 years and wheezing and atopy

	“Wheezing in the last year”^a^		“Only wheezing in the last year” (without atopy)^b^		“Wheezing + atopy in the last year”^b^		“Only atopy in the last year”^b^	
	Crude	Adjusted	Crude	Adjusted	Crude	Adjusted	Crude	Adjusted
18 years/22 years	OR (95% CI)	OR (95% CI)	OR (95% CI)	OR (95% CI)	OR (95% CI)	OR (95% CI)	OR (95% CI)	OR (95% CI)

**BMI obese (≥30 kg/m^2^**)	*P*=0.006	*P*=0.013	*P*=0.028	*P*=0.043	*P*=0.025	*P*=0.015	*P*=0.188	*P*=0.942
None	1.00	1.00	1.00	1.00	1.00	1.00	1.00	1.00
Only at 18 years	1.26 (0.60, 2.67)	1.28 (0.53, 3.11)	0.90 (0.21, 3.79)	0.71 (0.09, 5.42)	1.63 (0.68, 3.92)	1.83 (0.68, 4.99)	1.20 (0.72, 2.03)	1.29 (0.69, 2.42)
Only at 22 years	1.20 (0.84, 1.73)	1.19 (0.75, 1.88)	0.96 (0.52, 1.79)	0.73 (0.30, 1.75)	1.26 (0.81, 1.96)	1.63 (0.96, 2.76)	0.87 (0.68, 1.12)	1.19 (0.87, 1.63)
Both	1.92 (1.35, 2.73)	2.00 (1.32, 3.03)	2.14 (1.27, 3.60)	2.42 (1.31, 4.45)	1.63 (1.03, 2.59)	1.67 (0.96, 2.88)	0.85 (0.63, 1.15)	0.86 (0.60, 1.25)
**Total fat mass – BOD POD % in the third tertile**	*P*=0.226	*P*=0.108	*P*=0.567	*P*=0.095	*P*=0.128	*P*=0.092	*P*=0.845	*P*=0.377
None	1.00	1.00	1.00	1.00	1.00	1.00	1.00	1.00
Only at 18 years	0.96 (0.63, 1.46)	0.85 (0.50, 1.47)	0.80 (0.39, 1.63)	0.90 (0.37, 2.20)	1.15 (0.69, 1.93)	0.89 (0.46, 1.74)	1.16 (0.89, 1.51)	1.11 (0.80, 1.53)
Only at 22 years	0.89 (0.57, 1.38)	0.97 (0.57, 1.65)	0.63 (0.29, 1.39)	0.45 (0.14, 1.49)	1.11 (0.66, 1.87)	1.33 (0.74, 2.41)	1.07 (0.81, 1.40)	1.08 (0.77, 1.50)
Both	1.28 (0.98, 1.67)	1.42 (1.04, 1.95)	1.21 (0.80, 1.83)	1.69 (1.03, 2.78)	1.31 (0.93, 1.83)	1.38 (0.93, 2.06)	0.97 (0.80, 1.17)	1.10 (0.88, 1.38)
**Total fat mass – DXA % in the third tertile**	*P*=0.238	*P*=0.048	*P*=0.396	*P*=0.053	*P*=0.080	*P*=0.021	*P*=0.666	*P*=0.371
None	1.00	1.00	1.00	1.00	1.00	1.00	1.00	1.00
Only at 18 years	1.01 (0.64, 1.58)	0.98 (0.56, 1.73)	1.00 (0.49, 2.06)	1.25 (0.53, 2.97)	1.04 (0.59, 1.85)	0.87 (0.42, 1.83)	1.06 (0.79, 1.41)	1.05 (0.75, 1.48)
Only at 22 years	1.10 (0.71, 1.68)	1.16 (0.68, 1.99)	0.58 (0.24, 1.34)	0.50 (0.15, 1.67)	1.25 (0.76, 2.05)	1.43 (0.79, 2.62)	0.70 (0.52, 0.95)	0.81 (0.57, 1.17)
Both	1.34 (1.01, 1.77)	1.58 (1.14, 2.20)	1.33 (0.86, 2.08)	1.88 (1.11, 3.19)	1.36 (0.95, 1.94)	1.60 (1.06, 2.42)	1.02 (0.84, 1.24)	1.16 (0.92, 1.47)
**Trunk fat mass – DXA % in the third tertile**	*P*=0.324	*P*=0.064	*P*=0.595	*P*=0.086	*P*=0.078	*P*=0.014	*P*=0.876	*P*=0.108
None	1.00	1.00	1.00	1.00	1.00	1.00	1.00	1.00
Only at 18 years	0.95 (0.60, 1.48)	0.93 (0.53, 1.63)	0.75 (0.35, 1.59)	0.90 (0.37, 2.22)	0.99 (0.57, 1.73)	0.84 (0.42, 1.70)	0.85 (0.64, 1.14)	0.78 (0.55, 1.11)
Only at 22 years	1.03 (0.67, 1.58)	1.13 (0.67, 1.91)	0.35 (0.13, 0.98)	0.30 (0.07, 1.28)	1.35 (0.83, 2.18)	1.59 (0.90, 2.83)	0.77 (0.58, 1.03)	0.89 (0.63, 1.26)
Both	1.29 (0.97, 1.71)	1.54 (1.11, 2.16)	1.30 (0.84, 2.01)	1.84 (1.09, 3.10)	1.34 (0.94, 1.93)	1.65 (1.08, 2.51)	1.07 (0.88, 1.30)	1.26 (1.00, 1.59)
**FMI in the third tertile**	*P*=0.317	*P*=0.113	*P*=0.452	*P*=0.133	*P*=0.228	*P*=0.126	*P*=0.197	*P*=0.816
None	1.00	1.00	1.00	1.00	1.00	1.00	1.00	1.00
Only at 18 years	0.96 (0.62, 1.48)	0.78 (0.43, 1.40)	1.05 (0.54, 2.02)	1.11 (0.48, 2.57)	0.83 (0.46, 1.48)	0.58 (0.26, 1.29)	0.87 (0.65, 1.15)	0.88 (0.62, 1.25)
Only at 22 years	1.02 (0.66, 1.56)	1.01 (0.60, 1.69)	0.82 (0.40, 1.67)	0.66 (0.25, 1.73)	1.01 (0.60, 1.71)	1.14 (0.62, 2.10)	0.78 (0.58, 1.03)	0.82 (0.58, 1.17)
Both	1.27 (0.98, 1.66)	1.39 (1.02, 1.90)	1.23 (0.81, 1.86)	1.59 (0.97, 2.62)	1.24 (0.89, 1.74)	1.36 (0.92, 2.01)	0.92 (0.76, 1.10)	1.06 (0.85, 1.33)

**Notes:** Atopy, self-reported rhinitis, and/or eczema symptoms in the last year previous to the 22 years interview. Adjusted by gender, skin color, asset index (quintiles), education (years), maternal smoking during pregnancy, parents smoking during infancy, hospitalization during infancy, parents history of asthma, wheezing or bronchitis, household crowding, smoking trajectory 18–22 years, and physical activity trajectory 18–22 years.

a Logistic regression.

b Multinomial logistic regression (reference category: no wheezing and/or atopy reported).

**Abbreviations:** BMI, body mass index; DXA, dualenergy X-ray absorptiometry; FMI, fat mass index.
